# A time-space Bayesian regression model of rabies cases in the animal population of Kazakhstan (2013–2023)

**DOI:** 10.3389/fvets.2025.1640050

**Published:** 2025-11-11

**Authors:** Alberto Gomez-Buendia, Gulzhan Yessembekova, Ablaikhan Kadyrov, Yersyn Mukhanbetkaliyev, Eva Cerviño-Luridiana, Julio Alvarez, Andres M. Perez, Sarsenbay K. Abdrakhmanov

**Affiliations:** 1 VISAVET Health Surveillance Centre, University Complutense de Madrid, Madrid, Spain; 2 Departamento de Sanidad Animal, Facultad de Veterinaria, Universidad Complutense de Madrid, Madrid, Spain; 3 S. Seifullin Kazakh Agro Technical Research University, Astana, Kazakhstan; 4 Independent Researcher, Madrid, Spain; 5 Center for Animal Health and Food Safety, College of Veterinary Medicine, University of Minnesota, Saint Paul, MN, United States

**Keywords:** Bayesian, regression model, time-space, rabies, Kazakhstan, animals

## Abstract

**Introduction:**

Despite its endemic status and socioeconomic impacts, the spatial-temporal variation in rabies risk and its underlying determinants in Kazakhstan animal populations remain poorly understood. This study aimed to characterize the time-space dynamics of rabies in animal populations across Kazakhstan regions from 2013 to 2023 and identify the key drivers of transmission.

**Methods:**

Using a Bayesian hierarchical regression model with spatial and temporal random effects, we analyzed national surveillance data on rabies cases in livestock, companion animals, and wildlife, alongside sociodemographic and animal population variables.

**Results:**

The model revealed that higher median income (odds ratio [OR]: 1.18, 95% posterior predictive interval [PPI]: 1.06–1.31), the presence of rabies in wildlife (OR: 1.55, 95% PPI: 1.27–1.89), and companion animal rabies incidence (low: 1–5 cases/year, OR: 1.39, 95% PPI: 1.06–1.85; high: ≥6 cases/year, OR: 2.07, 95% PPI: 1.46–2.96) were associated with increased livestock rabies risk, while higher human population density correlated with reduced risk (OR: 0.68, 95% PPI: 0.5–0.9). Spatial analysis identified persistent high-risk zones in western Kazakhstan and lower risk in southern regions, driven by ecological and socioeconomic heterogeneity.

**Discussion:**

These findings highlight the relationship between wildlife reservoirs, domestic animal management, and socioeconomic factors in rabies transmission in Kazakhstan. By integrating these insights into national policy, Kazakhstan can advance toward the global target of eliminating dog-mediated human rabies deaths by 2030, serving as a model for Central Asia.

## 1 Introduction

Rabies is a severe, vaccine-preventable viral disease of the nervous system that affects both animals and humans (1, 2). The primary reservoirs for the rabies virus include wild and stray canids, certain species of rodents, and livestock (3). Rabies causes progressive and fatal inflammation of the brain and spinal cord. Once clinical signs appear, the case fatality rate approaches nearly 100% (4, 5).

Rabies kills approximately 59,000 people globally each year, although due to significant underreporting, the actual number of cases is likely much higher despite the availability of effective prevention tools: death from rabies can be prevented through timely post-exposure prophylaxis (PEP), which blocks the virus from entering the central nervous system. However, the use of PEP is costly. As of 2018, the estimated average cost of PEP was approximately $108 USD (including travel expenses and lost income), representing a significant financial burden for countries where individuals live on an average of $1–2 USD per day (6, 7).

Alarmingly, a large proportion (40%) of the victims are children under the age of 15. Domestic dogs are the primary source of human rabies deaths and have been considered responsible for approximately 99% of all human fatalities (8). For these reasons, rabies has been included in the WHO's 2021–2030 roadmap for neglected tropical diseases, which aims to build a global framework for the elimination of dog-mediated rabies and achieve zero human deaths from rabies worldwide by 2030 (6, 9).

Dog-mediated rabies has already been eliminated in Western Europe, Canada, the United States, Japan, South Korea, Singapore, and several Latin American countries (10–12). However, the disease remains a serious public health concern in more than 150 countries, primarily in Asia and Africa. In Eastern Europe and Central Asia, rabies is considered endemic (13) and can increase in the case of unfavorable circumstances. For instance, 63 human rabies cases were recorded in Ukraine between 1996 and 2020. According to the Ukraine Center for Public Health, 4,272 cases of rabies-infected animal bites were reported between 2023 and 2024, likely associated with the challenges in disease control associated with the social disruption suffered by the country in that year. In Azerbaijan, rabies is present in domestic animals and less commonly in wildlife, and between one and five human deaths due to rabies were reported annually between 2018 and 2023, while 13 rabies cases were reported in 2023 in Kyrgyzstan (14). According to the CDC, Russia was rated as a high-risk country for importing dog rabies into the United States, where 2,000–4,000 rabies cases in animals are reported each year (15). In China, rabies remains widespread among various species of wild, domestic, and farm animals. However, even though dog rabies hotspots persist, significant progress has been achieved, and human cases have dropped from 3,300 in 2007 to 516 in 2017 and approximately 202 in 2020 (16).

The Republic of Kazakhstan is considered an endemic territory for rabies, with 54 reported human rabies deaths since 2010 (with between one and 10 deaths reported each year, except in 2018, and no reported deaths in 2022 and 2023), according to the WHO (17). The first officially documented case of rabies in Kazakhstan dates back to 1914 in the Turgai region (18). Since then, the disease has been recorded in animals every year (19). Rabies causes significant economic losses, including livestock mortality, the cost of quarantine and preventive measures, trapping and managing stray dogs and cats, sterilization programs, regulation of wild carnivore populations, and diagnostic testing (7). In Kazakhstan, economic losses due to rabies have been estimated at 20.9 million USD annually, with about half of it attributed to PEP. In addition, vast efforts are also invested in animal vaccination, with an average of 4.7 million domestic animals vaccinated annually between 2013 and 2015, and 736,000 vaccine baits deployed for wildlife vaccination every year (20).

Current rabies control in Kazakhstan relies on passive surveillance, which includes reactive monitoring and emergency vaccination in response to detected cases. To illustrate the scale of these efforts, rabies vaccination in 2025 is planned to cover at least 5 million head of livestock, approximately 2.5 million companion animals, and up to 2 million wild carnivores, accounting for the country's vast territory and diverse climates. Wildlife vaccination follows WOAH recommendations, employing bait distribution, consumption monitoring, and tetracycline biomarker analysis, tailored to the local epizootic situation and density of susceptible wildlife. Key institutionalized measures encompass promoting responsible dog ownership, mass dog vaccination, control of fox populations, and management of stray animals. For human prevention, Post-Exposure Prophylaxis (PEP) is implemented using inactivated cell culture vaccines—such as COCAV (Russia/Kazakhstan), Verorab (France), Rabipur (Germany/India), and Rabivac (India)—administered according to a standard 5-dose schedule on days 0, 3, 7, 14, and 30.

As part of the ongoing efforts to control rabies in the animal reservoir, a zoning strategy based on the distinct epidemiological features of the disease in different regions of the country has been proposed to support disease control in Kazakhstan (21). However, time-space variation in disease risk and its potential association with certain variables with a heterogeneous spatial distribution, including animal populations and sociodemographic factors, has never been assessed in the country.

In this study, we fitted a multivariable Bayesian regression model to animal rabies incidence data from Kazakhstan (2013–2023) to characterize spatio-temporal variation in disease risk at the regional level. The model incorporated structured and unstructured random effects, as well as animal population and sociodemographic data, the influence of which has been demonstrated by Kabzhanova et al. (22). Integrating these covariates at the regional level into a single framework, our approach can capture not only complex space-time dependencies but also the influence of demographic factors on rabies risk. These results will extend previous rabies control efforts, ultimately contributing to the elimination of the disease across the country and in Central Asia.

## 2 Materials and methods

### 2.1 Background information

The Kazakhstan administrative division includes 14 oblasts (first-level administrative divisions, herein regions) and three cities of national significance (Astana, Almaty and Shymkent). The geography of the country is characterized by various landscapes, including extensive steppes, arid deserts, and significant water bodies such as Lake Balkhash. The country's climate is continental, characterized by significant temperature variations. In terms of demographics, Kazakhstan has a population of approximately 20.1 million, with a low population density (7 people/km^2^) and significant urbanization, since over 60% of the population resides in cities, with rural communities often engaged in agriculture and pastoralism.

### 2.2 Spatiotemporal analysis

The study relied on a national database provided by the Kazakh public authorities. This dataset included variables at the regional level aggregated by region and year, including rabies case counts in animals, animal population data and sociodemographic characteristics. The rabies case counts and animal populations were stratified in the categories livestock (cattle, camels, sheep and horses), companion animals (dogs and cats) and wildlife (wolves and foxes) due to the lack of information on case occurrence by species, in spite of the potential bias this could introduce in the analysis due to the different epidemiology of the disease in each animal species. The sociodemographic data included the total human population, the number of people living in urban and rural settings, the average annual income, and the total road length and road density in each region. The choice of two latter variables was influenced by the study of Kabzhanova et al. (22), which demonstrated that regions with significantly lower road density (e.g., Ulytau, Karaganda, Mangystau, Atyrau) tend to report fewer cases of rabies.

The spatiotemporal incidence of livestock rabies was assessed using a Bayesian hierarchical model with spatially structured and non-structured random effects as previously described (23). Briefly, the observed rabies in region *i* and year *j* was modeled as a Poisson distribution *O*_*ij*_ ~ *Poisson*(μ_*ij*_), where the log-linear predictor incorporated an offset for expected cases considering the annual median incidence of livestock rabies cases and assuming cases were distributed homogeneously in the country as a function of the exposed animal population (*E*_*i, j*_), the spatially structured (*S*_*i*_) and unstructured random effects (*U*_*i*_), and the available covariate terms (β_*k*_*X*_*ijk*_) as:


$$\begin{array}{l} \log\left(\mu_{i,j}\right)=\log\left(E_{i,j}\right)+\beta_{0}+S_{i}+U_{i}+\beta_{k}X_{ijk} \end{array}$$

Structured spatial effects were modeled using a conditional autoregressive (CAR) prior with adjacency defined by the Queen's Contiguity method, where regions sharing borders or corners were considered neighbors. Unstructured effects were assigned independent normal priors $U_{i}\;\sim \;N\left(\mu_{U_{i}},\;\sigma_{U_{i}}^{2}\right)$. Both were assigned gamma distributions τ ~ *Gamma*(1, 0.01) as hyperprior distributions on the inverse variance parameters. All the coefficients for the available covariates were set to follow weakly informative Normal prior distributions as β_*k*_ ~ *N*(0, ~0.01).

Prior to model fitting, we assessed potential multicollinearity among the selected covariates by fitting a standard linear regression model with the same set of predictors and calculating the variance inflation factor (VIF) through the package “performance”. All continuous covariates (including animal population variables and sociodemographic characteristics) except the urban-to-rural ratio distribution were standardized using z-score transformation (centered by subtracting the mean and scaled by dividing by the standard deviation) using the “scale” function in R. This procedure was implemented to minimize the convergence issues arising from the disparate variable magnitudes. Urban and rural population distribution was as expressed a ratio (proportion of the urban/rural residents relative to the total regional population). Companion and wildlife rabies case counts were evaluated as discrete variables and, additionally, as categorical variables to account for non-linear associations with livestock rabies risk. Several categorisations were explored, such as presence or absence of cases, or categorization into quartiles. Ultimately, however, companion animal case counts were categorized as ‘none' (0 cases), ‘low' (1–5 cases) or ‘high' (≥6 cases) based on the terciles of the empirical distribution of annual cases (2020–2024) based on the better model fit. Wildlife rabies case counts were dichotomized as ‘absent' (0 cases) or ‘present' (≥1 case) given the low numbers recorded.

For variable selection, a set of univariable models including the spatially structured and non-structured random effects were first fitted to the livestock case counts for each of the available covariables. Variables with exponentiated coefficients whose 95% posterior predictive intervals (PPI) excluded 1 were further considered in the multivariable analysis. In the multivariable analysis, DIC was used to select the best model. To validate the adequacy of the Poisson distribution assumption in the model, we conducted posterior predictive checks by generating simulated livestock case count data from the model and comparing these to the observed counts.

As a sensitivity analysis, we fitted a simplified hierarchical Bayesian model excluding the spatially structured random effects. The final model incorporated only unstructured area-level random effects, along with relevant covariates and temporal structure. This allowed us to assess the robustness of our results to the inclusion of spatial autocorrelation. The analysis was conducted in R (24) using the “R2OpenBUGS” package (25) to interface with OpenBUGS for Bayesian inference via Markov Chain Monte Carlo (MCMC) sampling. Three MCMC chains were run for 15,000 iterations with a ‘burn-in' of 1,000 iterations and posterior distributions were calculated after thinning every 10 iterations. Convergence was assessed visually using the ‘mcmcplots' package (26) and formally by the Gelman–Rubin statistic (27). Spatial adjacency matrices and weights were constructed using geographic boundary data from the R package “geokz” (28) using “spdep” package (29).

## 3 Results

**Figure 1:**
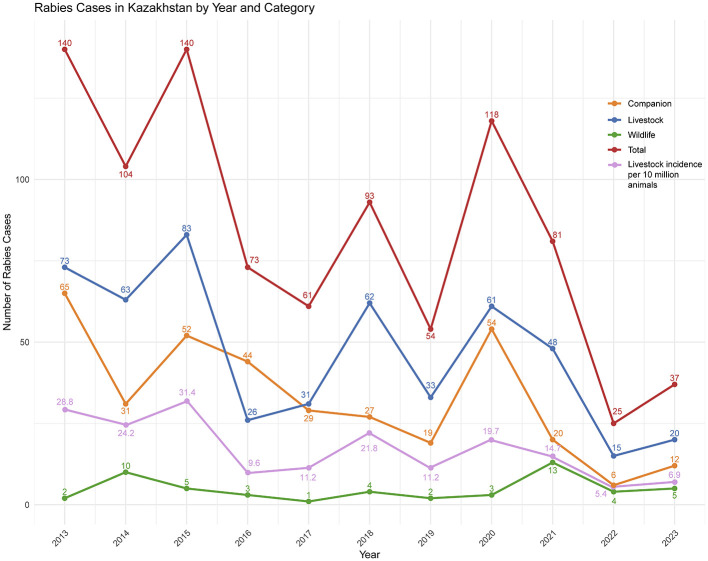
Annual cases of rabies in livestock, companion animals and wildlife.

**Figure 2:**
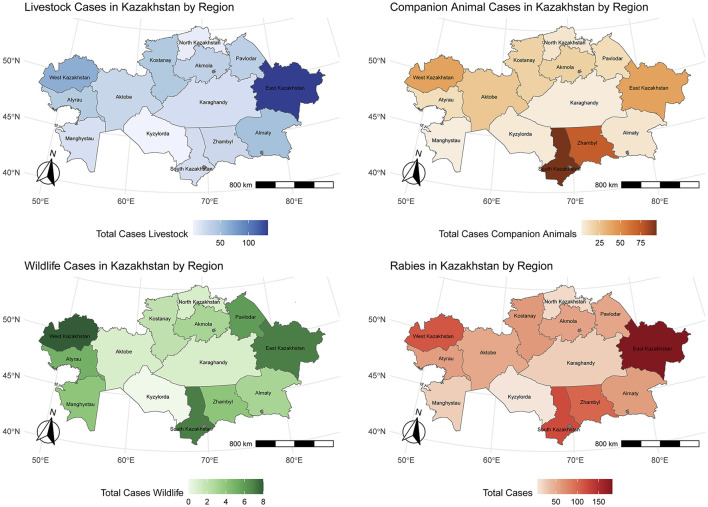
Accumulated cases of rabies in livestock, companion animals and wildlife by region from 2013 to 2023.

During the 10-year period of the study, 926 cases of rabies in animals were reported in Kazakhstan. Of those, 515 were in livestock animals (55.6%), 359 in companion animals (38.8%) and 52 in wildlife (5.6%). The case counts reported varied annually (Figure 1) between regions (Figure 2). The annual mean incidence in livestock over the study period was 16.8 cases (min = 5.4, max = 31.4) per 10 million animals (Figure 1).

All VIF were below 2, suggesting a lack of multicollinearity between covariates. The spatiotemporal Bayesian hierarchical multivariable model revealed associations between livestock rabies incidence and several covariates after adjusting for structured spatial effects and spatially unstructured heterogeneity. Median income exhibited a positive association with rabies risk, with a posterior median odds ratio (OR) of 1.18 (95% PPI: 1.06–1.31), indicating that regions with higher median income had elevated rabies incidence in livestock. Conversely, the total population size was negatively associated with disease frequency, with a median OR of 0.68 (95% PPI: 0.5–0.9). Companion and wildlife rabies case counts were included as categorical variables based on the improved model fit. Wildlife rabies presence (≥1 case) in a region was associated with increased livestock rabies risk (median OR: 1.55; 95% PPI: 1.27–1.89) while detection of rabies in companion animals was associated with a different increase in disease risk in livestock depending on the number of cases recorded: low frequency of rabies in companion animals (1–5 cases in a year) led to a median increase in the odds of disease of 1.39 (95% PPI: 1.06–1.85) in livestock compared to the situation in which no disease was detected in this animal category, while high-frequency years in a region (≥6 cases) was linked to a larger increase in rabies risk (median OR: 2.07; 95% PPI: 1.46–2.96). Although the 95% PPI from the covariate representing the wolf population density included 1 (median OR: 1.05; 95% PPI: 0.92–1.2). The inclusion of this covariate improved the model fit, as evidenced by a reduction in the DIC from 605.2 to 351.3 (calculated as a difference between nested models, i.e. baseline model vs. model with spatially structured random effects) and hence was maintained in the model (Table 1).

**Table 1:** Univariable and multivariable model results for livestock rabies risk.

**Variable**	**Univariable OR (95% PPI)**	**Saturated multivariable model OR (95% PPI)**	**Final multivariable model OR (95% PPI)**
**Wildlife rabies**			
No cases	REF^a^	–	–
≥1 case per year	1.65 (1.10–2.13)	1.56 (1.27–1.91)	1.55 (1.27–1.89)
**Companion animal rabies**			
No cases	REF^a^	–	–
1–5 cases per year	1.34 (1.04–1.46)	1.40 (1.06–1.85)	1.39 (1.06–1.85)
≥6 cases per year	2.12 (1.55–2.37)	2.09 (1.47–3.00)	2.07 (1.46–2.96)
Wolf population	1.07 (0.93–1.23)	1.06 (0.91–1.21)	1.05 (0.92–1.20)
Median income	1.12 (1.00–1.16)	1.16 (1.03–1.30)	1.18 (1.06–1.31)
Median road density	1.07 (0.82–1.17)	1.04 (0.79–1.38)	–
Total population	0.6 (0.49–0.73)	0.66 (0.49–0.90)	0.68 (0.50–0.90)
Urban-to-rural ratio	1.10 (0.79–1.24)	1.11 (0.80–1.58)	–
DIC		590.4	351.3

^a^Reference category.

**Figure 3:**
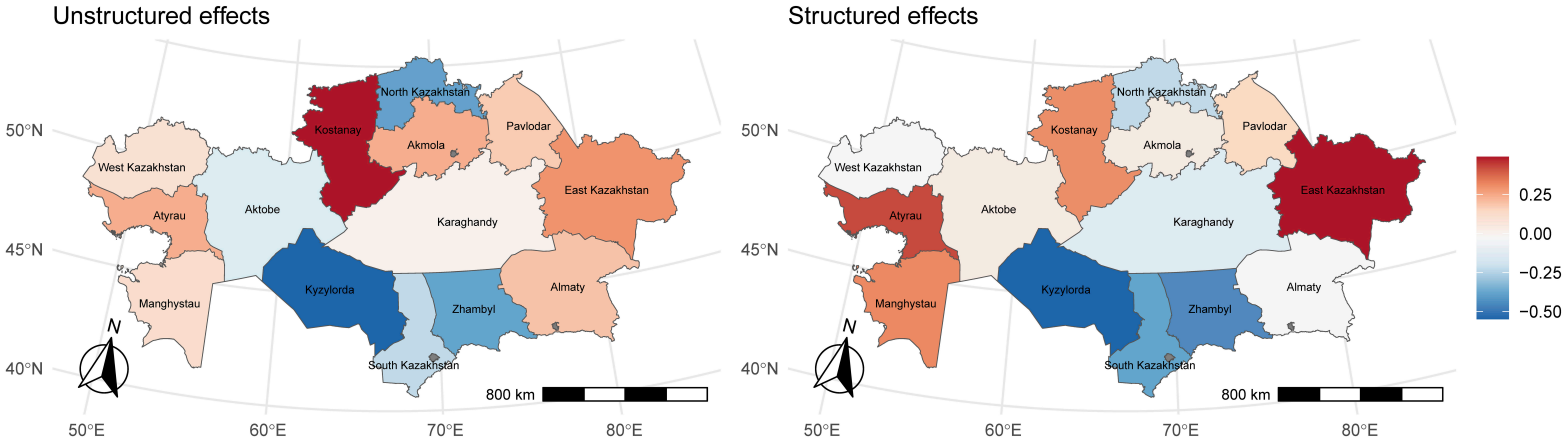
Posterior median estimates of unstructured and structured effects by region.

The median posterior values estimated for the spatially structured and unstructured random effects were similar, suggesting a comparable importance of spatial and non-spatial heterogeneity (Figure 3). According to both random effects, the regions in the south of the country (Turkestan, Jambyl, and Kyzylorda) were consistently exposed to a lower risk of rabies (spatially structure posterior median: 0.68, 0.64, and 0.58, respectively), while those in the east and west (Mangystau, Kostanay, Atyrau, and East Kazakhstan) experienced a higher risk (spatially structure posterior median: 1.37, 1.38, 1.55, 1.66, respectively), and a higher heterogeneity was observed in other parts of the country.

The mean and standard deviation of the observed aggregated counts aligned closely with the distributions of the simulated values indicating that the Poisson structure appropriately fitted the observed data (Supplementary material 1).

The sensitivity analysis showed that removing the spatially structured random effects did not substantially alter the estimated coefficients or their associated probability intervals. However, we retained the full spatiotemporal model as the final version, as it provides a more comprehensive representation of potential spatial heterogeneity given the known spatial distribution of rabies cases. Additionally, the model with spatially structured effects had a substantially lower DIC (351.3 vs. 662.3), supporting its selection as the final model (Supplementary material 2).

## 4 Discussion

Rabies is a fatal zoonosis that remains a significant economic and public health concern, yet it is entirely preventable and ultimately eradicable (30, 31). To achieve the WHO's 2030 elimination target (6), data-driven strategies are required to address its complex spatiotemporal dynamics at a national and regional level. In order to inform targeted control efforts in Kazakhstan, we conducted a spatiotemporal Bayesian analysis of a decade-long reported rabies cases, which allowed capturing large-scale trends at the regional level.

Our analysis revealed a slightly higher livestock rabies risk in regions with a higher median income like East Kazakhstan. This result may appear counterintuitive, but it may also reflect economic disparities in animal husbandry practices. In Kazakhstan, higher-income regions tend to have larger livestock populations, which could increase the likelihood of transmission as well as favor the contact between livestock and reservoir species, as in the case of other countries with strong pastoralist traditions, like Mongolia (32). In addition, lower-income areas tend to prioritize subsistence farming over commercial livestock production, which may reduce exposure, same as in farms in Ethiopia (33). This finding is consistent with other studies in Kazakhstan, which indicate that economic losses from rabies disproportionately affect regions with intensive livestock sectors. Furthermore, higher-income regions may implement more effective passive surveillance and have improved reporting infrastructures. This can result in increased case detection and an apparent rise in incidence (20).

The negative correlation between total population size and rabies risk may be attributable to urbanization trends. Over 60% of the Kazakhstan population lives in cities, where veterinary services, including vaccination and stray animal control, are more accessible. Rural areas, despite having lower population density, often face logistical challenges in implementing vaccination campaigns, which can perpetuate enzootic transmission (34–36). This finding aligns with the conclusions of other studies, which identified urbanization as a protective factor due to its capacity for rapid response and the administration of PEP, and increased public awareness, thereby reducing the risk of transmission in peri-urban livestock farms (37–39). However, when dog vaccination coverage is low urban areas can still experience persistent rabies transmission (40).

The strong association between the presence of rabies in wildlife and the risk to livestock highlights the potential role of sylvatic cycles in maintaining disease transmission. In Kazakhstan, wolves and foxes are the main reported wildlife hosts, particularly in the western and eastern regions, where landscapes support their ecological presence. These are known to act as reservoirs of the rabies virus and may spillover into domestic animal populations. Previous research in countries such as Russia, China and Mongolia has shown spatial co-occurrence between cases of rabies in (mainly) foxes and wolves, with cases in livestock, suggesting a sustained interface that supports cross-species transmission (32, 41, 42). Our results are a clear indication of the need for targeted surveillance and preventive measures in wildlife populations, such as oral vaccination programmes, which have proven effective in similar ecological conditions (43, 44). However, the relatively low number of cases reported in our dataset (only 52 cases over a decade) may reflect limitations in wildlife surveillance rather than true incidence, indicating that the contribution of wildlife to rabies persistence could be substantially underestimated.

Furthermore, companion animal rabies was also associated with an increased risk of rabies in livestock. This suggests the presence of overlapping transmission networks between stray dogs, cats, and livestock, particularly in the densely populated southern Kazakhstan. These findings have also been reported elsewhere (22, 45), where significant clusters in the southern regions driven by domestic animal cycles were identified. Thus, effective stray dog management is critical in the fight against rabies, as unvaccinated dogs can act as a bridge between wildlife, livestock, and ultimately human populations (46).

Spatial random effects revealed certain regional disparities once the effect of other covariates had been taken into account, with a higher risk in western Kazakhstan and a lower risk in the south. These results show the need to establish region-based surveillance in order to control rabies transmission. In a previous study, Abdrakhmanov et al. (21), analyzing historical data from 2003 to 2014, suggested that it would be advisable to apply zoning measures for rabies control. Similar to our results in terms of risk, they classified western regions as high-risk endemic zones due to favorable ecological conditions for wildlife reservoirs, including vast steppes and limited vaccination coverage. On the other hand, southern regions, despite high human and livestock densities, will benefit from stricter biosecurity measures in commercial farms and veterinary centers. Notably, the spatial risk patterns observed in our study mirror those identified a decade earlier, suggesting persistent geographical trends in rabies distribution.

Kazakhstan supports the Global Strategic Plan “Zero by 30” and has developed a national rabies elimination plan incorporating the “One Health” concept and multisectoral collaboration. This initiative focuses on improving PEP access, promoting bite prevention awareness, and expanding dog vaccination coverage to reduce human exposure risk. To achieve the WHO's 2030 elimination goal, policy frameworks must evolve through legislative reforms including mandatory dog vaccination laws and intersectoral collaboration (6), drawing inspiration from Latin America's successful centralized campaigns (10, 47). Public awareness programs targeting rural communities, particularly children who comprise 40% of global rabies deaths (6), could further reduce exposure risks. Kazakhstan stands out in Central Asia for its robust data analysis compared to southern neighbors: while Turkmenistan and Uzbekistan lack peer-reviewed rabies studies, and Kyrgyzstan has only one zoonosis burden review (48), Tajikistan's two studies include genomic characterization (49) and epidemiological analysis showing declining human cases linked to vaccination programs and livestock-canine transmission patterns (50) that mirror our findings.

Compared with previous studies in Kazakhstan that identified distinct spatial clusters of rabies using the spatial scan statistics (22, 51, 52) and proposed risk-based zoning using environmental predictors (21), our Bayesian hierarchical approach quantifies how specific demographic (e.g., income disparities, urbanization), animal population (e.g., wildlife spillover, companion animal incidence), and spatial dependency factors interact to modulate livestock rabies risk across regions. This study goes beyond cluster detection by explaining why disparities persist, a finding supported by recent Knowledge, Attitude, and Practices (KAP) studies that reveal significant gaps in rabies awareness and risky livestock management practices among farmers in high-risk regions, directly influencing exposure and transmission dynamics (53). Consequently, our analysis provides a transferable framework for Central Asian nations where similar sociodemographic heterogeneity may modulate zoonotic risk.

While our model advances our understanding of rabies dynamics, several limitations should be considered. The study relies on passive surveillance data, which is prone to underreporting, particularly in wildlife and in remote or sparsely populated regions. Surveillance activities are primarily conducted by regional veterinary services, and their capacity and reporting intensity may vary across regions. It has been noted that some northern and central regions consistently report fewer cases, which may reflect limited detection rather than true absence of disease (20). Consequently, observed spatial patterns may be influenced not only by ecological or epidemiological factors but also by differences in surveillance infrastructure. Our Bayesian modeling framework helps to mitigate some of this uncertainty by incorporating unstructured random effects, which absorb region-specific heterogeneity, including potential underreporting bias, leading to more robust estimates of the association between covariates and disease risk. Nonetheless, the model cannot fully correct for unmeasured reporting biases, and the results should be interpreted as reflecting the patterns within the reported data Additionally, we lacked information on rabies cases differentiated by species, as it was only by broader groups. This clearly limits our analysis, as the consequences in terms of management measures and costs for one species or another (e.g., cows vs. sheep) are not the same. Additionally, the dynamics of rabies transmission between different animal populations, such as dogs (major reservoirs of rabies that can transmit it to humans and other animals) and cats (most commonly accidental hosts with limited epidemiological relevance), differ considerably.

It is worth mentioning that we could not include covariates such as bat-related rabies dynamics in our analysis. Between 2020 and 2022, the rabies virus was detected in bats across six out of nine sample regions in Kazakhstan. Certain regions, such as Atyrau and North Kazakhstan, had prevalences up to 12%, including historically high-risk regions. While our study focused on terrestrial cycles, bat-borne rabies may constitute an understudied transmission route. Phylogenetic analysis has placed one bat-derived sequence in the Central Asia subclade, suggesting a potential cross-species spillover from terrestrial hosts (54). Although the focus has been established on terrestrial-based cycles, it would be advisable to evaluate the impact of bats on the rabies transmission network in Kazakhstan, as has been done successfully in Latin American countries (55). Active surveillance methodologies for chiropteran reservoirs, such as targeted capture and sampling of bats in roosts and migratory corridors, coupled with enhanced passive surveillance of grounded or neurologically abnormal bats, are critical to accurately assess the prevalence and distribution of bat-borne rabies variants (56, 57). Future surveillance measures and spatiotemporal studies should integrate this, together with genomic data to assess transmission networks, risk corridors and evaluate potential problems in vaccination (19, 58).

This study demonstrates the usefulness of Bayesian spatiotemporal models to unravel the complex epidemiology of rabies and to inform precision control strategies that combine socioeconomic and animal-related factors. Implementation of zoning strategies may be key for disease control in the future, as shown by the agreement of our results, performed with a more complete dataset, with previous studies. The interaction of many agents involved in rabies transmission and maintenance makes it difficult for Kazakhstan to achieve the targets set. However, policy reforms, implementation of WHO-recommended measures such as an appropriate and consistent combination of oral wildlife vaccination, mass dog vaccination, and enhanced PEP accessibility, combined with scientific research and molecular epidemiological studies, position Kazakhstan to achieve the WHO's 2030 elimination goal while serving as a model for Central Asia (59).

## Data Availability

The raw data supporting the conclusions of this article will be made available by the authors, without undue reservation. Requests to access the datasets should be directed to Sarsenbay K. Abdrakhmanov (s_abdrakhmanov@mail.ru).
