# Supplementary material for: A time-space Bayesian regression model of rabies cases in the animal population of Kazakhstan (2013–2023)
Source: Front Vet Sci. 2025 Nov 11;12:1640050. doi: 10.3389/fvets.2025.1640050 (PMC12643857; doi:10.3389/fvets.2025.1640050)
Supplement: Supplementary file 1 [file Supplementary_file_1.docx]

**Supplementary Material 1: Posterior predictive checking**

**
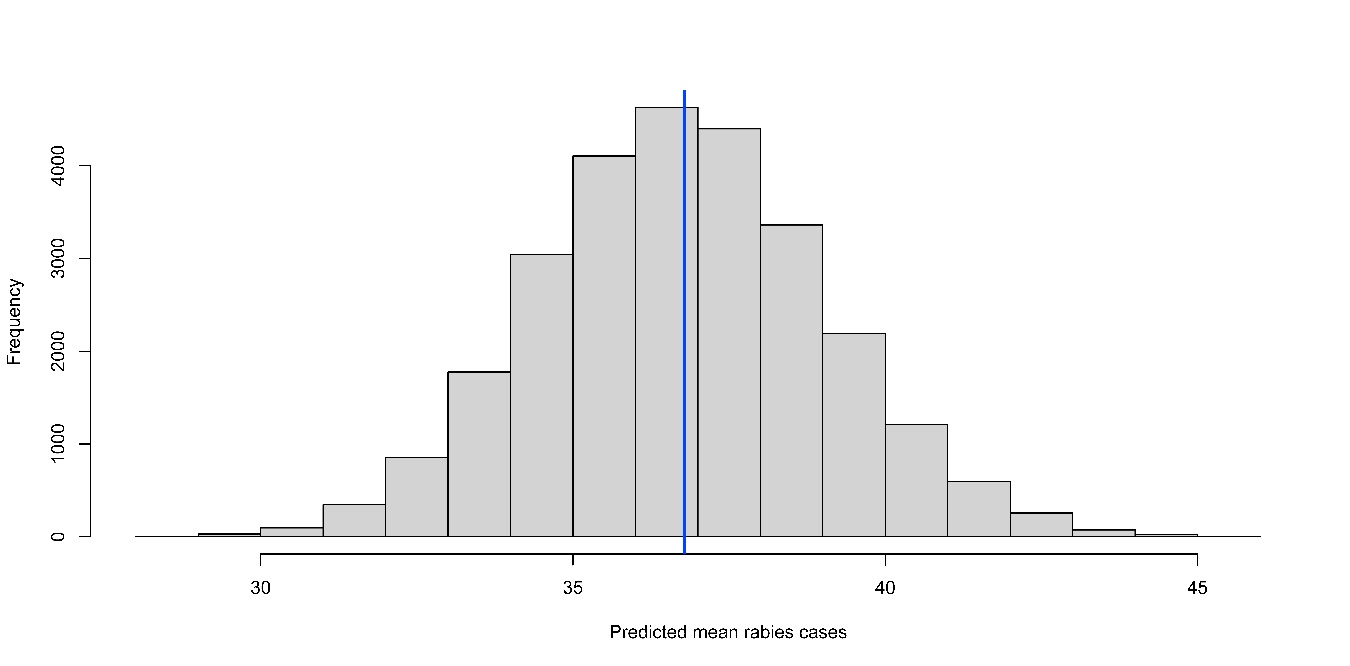
 Supplementary Figure 1.**

**
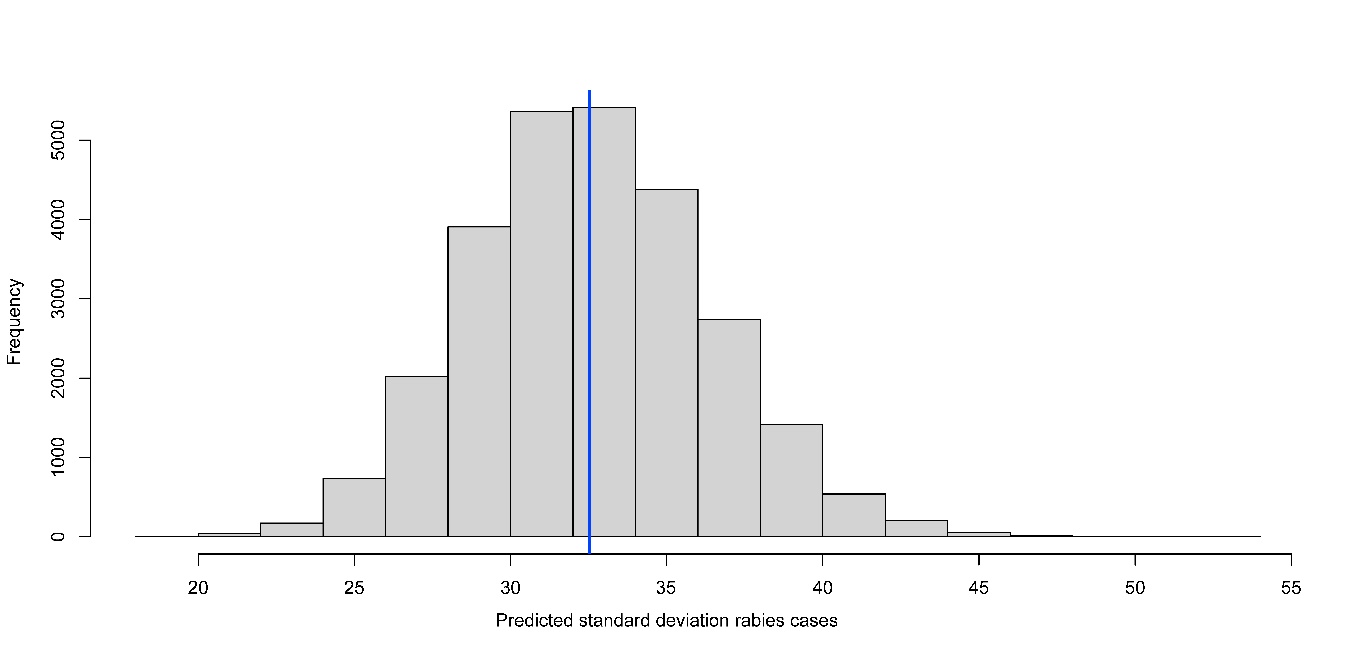
**

**Supplementary Figure 2.**

The mean (blue line in Supplementary Figure 1) and standard deviation (blue line in Supplementary Figure 2) of the observed livestock rabies incidence data compared against the simulated Poisson distributions of rabies cases.
